# Culprit vessel revascularization first with primary use of a dedicated transradial guiding catheter to reduce door to balloon time in primary percutaneous coronary intervention

**DOI:** 10.3389/fcvm.2022.1022488

**Published:** 2022-10-28

**Authors:** Jincheng Guo, Guozhong Wang, Zixuan Li, Zijing Liu, Yujie Wang, Senhu Wang, Yuntao Wang, Yongxia Wu, Haotian Wang, Yuping Wang, Libin Zhang, Qi Hua

**Affiliations:** ^1^Division of Cardiology, Beijing Luhe Hospital, Capital Medical University, Beijing, China; ^2^Division of Cardiology, Beijing Xuanwu Hospital, Capital Medical University, Beijing, China

**Keywords:** percutaneous coronary intervention, myocardial infarction, door-to-balloon time, culprit vessel, ST-elevation myocardial infarction

## Abstract

**Background:**

The effect of a single transradial guiding catheter (STGC) for culprit vessel percutaneous coronary intervention (PCI) first on door-to-balloon (D2B) time remains unclear.

**Materials and methods:**

Between February 2017 and July 2019, 560 patients with ST-elevation myocardial infarction (STEMI) were randomized into either the STGC group (*n* = 280) or the control group (*n* = 280) according to direct culprit vessel PCI with a STGC. In the STGC group, a dedicated transraidal guiding catheter (6F either MAC3.5 or JL3.5) was used for the treatment of electrocardiogram (ECG)-guided culprit vessel first and later contralateral angiography. In the control group, a universal diagnostic catheter (5F Tiger II) was used for complete coronary angiography, followed by guiding catheter selection for culprit vessel PCI. The primary endpoint was D2B time, and the secondary endpoint included catheterization laboratory door-to-balloon (C2B), procedural, fluoroscopy times, and major adverse cardiac events (MACE) at 30 days.

**Results:**

The median D2B time was significantly shorter in the STGC group compared to the control group (53.9 vs. 58.4 min; *p* = 0.003). The C2B, procedural, and fluoroscopy times were also shorter in the STGC group (C2B: 17.3 vs. 24.5 min, *p* < 0.001; procedural: 45.2 vs. 49.0 min, *p* = 0.012; and fluoroscopy: 9.7 vs. 11.3 min, *p* = 0.025). More patients achieved the goal of D2B time within 90 min (93.9% vs. 87.1%, *p* = 0.006) and 60 min (61.4% vs. 51.1%, *p* = 0.013) in the STGC group. Radial artery perforation (RAP) was significantly reduced in the STGC group compared with the control group (0.7% vs. 3.2%, *P* = 0.033). MACE at 30 days was similar (2.5% vs. 4.6%, *P* = 0.172) between the two groups.

**Conclusion:**

ECG-guided immediate intervention on culprit vessel with a STGC can reduce D2B, C2B, procedural, and fluoroscopy times (ECG-guided Immediate Primary PCI for Culprit Vessel to Reduce Door to Device Time; NCT03272451).

## Introduction

Primary percutaneous coronary intervention (PCI) is currently the preferred method of treatment for acute ST-elevation myocardial infarction (STEMI) when therapy can be performed in a timely fashion (1). Door-to-balloon (D2B) time is a well-established metric of primary PCI care quality. A shorter D2B time is associated with lower mortality in patients with STEMI undergoing primary PCI (2, 3). Many strategies have been proposed to reduce the time from symptom onset to arrival at the catheterization laboratory (4), but few studies have focused on catheterization laboratory strategies.

Compared to femoral access, radial access reduces mortality, major adverse cardiovascular events, and major bleeding in patients with STEMI undergoing PCI (5). However, some studies have shown that radial access is associated with a longer D2B time or lidocaine administration to the first device time (6–8), which offsets part of the radial access advantage.

One way to improve D2B time is to use a single transradial guiding catheter (STGC) for both coronary angiography and culprit vessel PCI, but there have been few studies regarding using a STGC to reduce D2B time (9–11). Another way to shorten the D2B time is by direct PCI without diagnostic angiography of non-culprit vessels (12); however, these were not randomized studies. It is unknown whether the combined STGC and culprit vessel PCI first strategy affects the D2B time.

In this single-center, prospective, randomized study, we compared two strategies: electrocardiogram (ECG)-guided immediate intervention on culprit vessel with a STGC versus complete diagnostic coronary angiography followed by culprit vessel PCI, to assess D2B time.

## Materials and methods

### Population and study protocol

The RAPID II (ECG-guided Immediate primary PCI for Culprit vessel to Reduce Door to Device Time) trial was a prospective, randomized, controlled, single-center study carried out at the Beijing Luhe Hospital between February 2017 and July 2019, wherein 751 patients were screened for participation in the study. Of these, 560 (74.6%) patients with STEMI who underwent primary PCI were enrolled in the study. The inclusion criteria were medical consultation <12 h after symptom onset, chest pain lasting ≥ 30 min, ST-elevation ≥ 1 mm in ≥ 2 adjacent ECG leads, or a new left bundle branch block. Patients were ineligible if they were on dialysis, had previous coronary bypass surgery, had received fibrinolytic therapy, or had an absent radial pulse. This study was approved by the ethics committee of our hospital, and all patients provided written informed consent before randomization.

**FIGURE 1:**
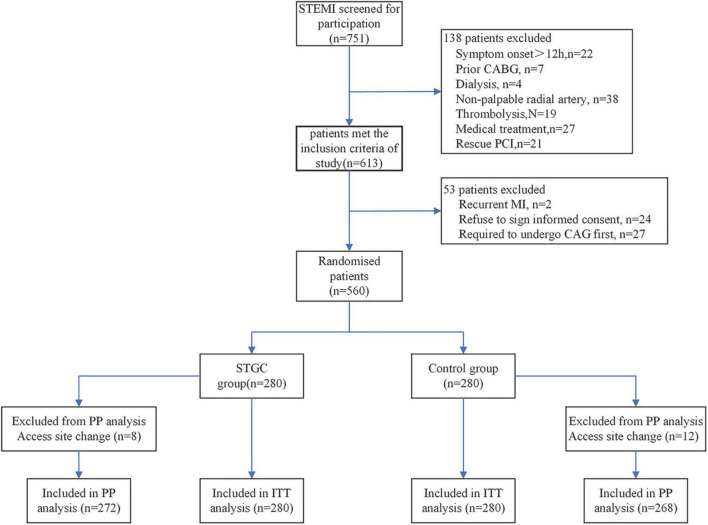
Trial flowchart. CABG, coronary artery bypass graft; ITT, intention-to-treat; PP, per-protocol.

Patients were randomized into two groups in a 1:1 ratio in blocks of four (with sealed and opaque envelopes). The STGC group (*n* = 280) underwent ECG-guided culprit vessel PCI using a STGC, either 6F MAC 3.5 (Medtronic Corporation, Minneapolis, MN, USA), or JL 3.5 (Medtronic Corporation, Minneapolis, MN, USA), followed by contralateral angiography using the same guiding catheter. JL3.5 guiding catheter manipulation technique for RCA (Supplementary Video 1) and MAC 3.5 guiding catheter for both LCA and RCA (Supplementary Videos 2A,B). If the initial STGC failed to engage the culprit vessel, contralateral angiography with the same STGC was allowed before changing the guiding catheter for the culprit vessel PCI. The control group (*n* = 280) underwent complete coronary angiography with a 5F Tiger II (Terumo Corporation, Tokyo, Japan) diagnostic catheter, followed by culprit vessel PCI (Figure 1).

### Percutaneous coronary intervention procedure

All patients were pretreated with a loading dose of 300 mg aspirin and a P2Y12 inhibitor (clopidogrel 600 mg or ticagrelor 180 mg) before PCI. The right radial artery was the default access. After successful 6-Fr sheath insertion, Heparin and bivalirudin are acceptable anticoagulants. Unfractionated heparin (70–100 IU/kg body weight) was administered intravenously and then guided by activated clotting time results, aiming to maintain an activated clotting time of >250 s or 200–250 s with bailout use of a GP IIb/IIIa inhibitor. For patients who received bivalirudin, an intravenous bolus of 0.75 mg/kg followed by an infusion of 1.75 mg/kg/h for 4 h after the procedure was recommended. The choice of the PCI device and use of GP IIb/IIIa inhibitor was left to the discretion of the operator. The arterial sheath was removed immediately after PCI, and hemostasis was achieved with external compression using a TR band (Terumo, Tokyo, Japan) for 6 h. The procedures were performed by four experienced interventional cardiologists who performed transradial PCI in more than 200 cases annually. Radial artery angiography should be performed immediately if unexpected resistance is felt during the advancement of the guidewire or catheter for the early detection of radial artery perforation (RAP). The patients were discharged on dual antiplatelet therapy for at least 12 months.

### Definitions and outcome measurement

The procedural time was defined as the time from local anesthesia to the withdrawal of the last catheter. The D2B time refers to the interval between hospital arrival and the first device (balloon, aspiration catheter, or direct stenting) used in the culprit artery. The C2B time was the interval between catheterization laboratory arrival and the first device used in the culprit artery. Puncture-to-balloon time was the interval between the access puncture and the first device used in the culprit artery. New onset atrial fibrillation (AF) was considered in patients with AF during hospitalization but not documented in previous medical history. Procedural success was defined as TIMI 3 flow in the culprit vessel and residual stenosis of <20% in the treated segment at the end of the procedure, without major clinical complications (e.g., death, myocardial infarction, and emergency coronary artery bypass graft) during hospitalization. The STGC group underwent coronary angiography for both the left coronary artery, including the left anterior descending artery (LAD), left circumflex artery (LCX), and right coronary artery (RCA), and complete culprit vessel PCI was defined as STGC success. Major adverse cardiac events (MACE) included cardiac death, reinfarction, and ischemia-driven target vessel revascularization. RAP was defined as persistent extravascular loss and accumulation of contrast medium through the vessel wall, as demonstrated by angiography.

The primary endpoint was the D2B time. The secondary endpoints included C2B, procedural, fluoroscopy times, and MACE at 30 days follow-up.

### Statistical analysis

All statistical tests were performed using SPSS (version 17.0; Chicago, IL, USA). Continuous variables are expressed as mean ± standard deviation or median [interquartile range (IQR)] and were compared using the Student’s *t*-test or Mann–Whitney test, where appropriate. Categorical variables were indicated as absolute numbers and percentages and were compared using the Pearson χ^2^ test or, if the number expected was <5, using the Fisher exact test. A 2-tailed *p* < 0.05 was considered significant. The primary analysis was based on the intention-to-treat principle. A per-protocol analysis (assessment only in cases without access site changes) was performed.

The sample size calculation was performed using a superiority design with an assumed D2B time difference (5 min) between the STGC group and the control group (9, 13). To detect a difference of 5 min in the D2B time with a power of 90% and an α error of 0.5, considering a dropout rate of 10%, the number required was calculated to be at least 560 patients.

## Results

### Baseline characteristics

A total of 560 patients who presented with STEMI during the study period were analyzed, with 280 in each group (STGC group vs. control group). There were 456 men and 104 women, with an average age of 59.4 ± 12.5 years. The baseline characteristics of the patients who underwent randomization are shown in Table 1. There were no significant differences in baseline clinical characteristics between the two groups.

**TABLE 1:** Demographics and baseline characteristics.

	STGC group	Control group	*P*-value
	(*n* = 280)	(*n* = 280)	
Age (yrs)	59.8 ± 12.7	58.9 ± 12.2	0.41
Male, *n* (%)	225 (80.4)	231 (82.5)	0.51
Diabetes mellitus, *n* (%)	90 (32.1)	78 (27.9)	0.27
Hypertension, *n* (%)	169 (60.4)	175 (62.5)	0.60
Hypercholesterolemia, *n* (%)	95 (33.9)	89 (31.8)	0.59
Present smoking, *n* (%)	159 (56.8)	176 (62.9)	0.13
History of stroke, *n* (%)	37 (13.2)	42 (15.0)	0.54
Previous MI, *n* (%)	21 (7.5)	18 (6.4)	0.62
Previous PCI, *n* (%)	22 (7.9)	24 (8.6)	0.76
History of AF, *n* (%)	4 (1.4)	1 (0.4)	0.37
New onset AF, *n* (%)	5 (1.8)	13 (4.6)	0.09
**Killip class on admission, *n* (%)**			0.98
I	232 (82.9)	232 (82.9)	
II	21 (7.5)	20 (7.1)	
III	6 (2.1)	5 (1.8)	
IV	21 (7.5)	23 (8.2)	
Systolic blood pressure, mmHg	120.1 ± 23.4	119.2 ± 23.8	0.63
Diastolic blood pressure, mmHg	77.4 ± 16.5	79.5 ± 17.1	0.13
Heart rate, beats/min	76.8 ± 18.6	77.2 ± 19.0	0.84
Peak CK (U/L)	2768.2 ± 2294.7	2731.3 ± 2082.1	0.843
Peak CK-MB (U/L)	206.2 ± 142.9	207.5 ± 145.7	0.92
**In-hospital medication, *n* (%)**			
Aspirin	280	280	1.00
Clopidogrel	133 (47.5)	131 (46.8)	0.87
Ticagrelor	147 (52.5)	149 (53.2)	0.87
Statin	268 (95.7)	266 (95.0)	0.69
ACEI/ARB	178 (63.6)	175 (62.5)	0.79
Beta-blocker	193 (68.9)	186 (66.4)	0.53
OAC	3 (1.1)	5 (1.8)	0.72

Values are mean ± SD, *n* (%). ACEI, angiotensin-converting enzyme inhibitor; AF, atrial fibrillation; ARB, angiotensin receptor blocker; CK, creatinine kinase; MI, myocardial infarction; PCI, percutaneous coronary intervention; OAC, oral anticoagulant.

### Procedural characteristics and angiographic results

No significant differences were noted between the two groups with regard to the number of diseased vessels, distribution of culprit vessels, TIMI flow before PCI, aspiration catheter use, administration of GP IIb/IIIa inhibitor, and contrast medium volume. Compared with the control group, the STGC group had less total number of catheters (1.2 ± 0.5 vs. 2.2 ± 0.6, *p* < 0.001).

During diagnostic coronary angiography, in the STGC group, initial guiding catheter engagement failed in eight cases (3 LCA and 5 RCA), of which three cases with culprit vessels failed. In the control group, the Tiger II diagnostic catheter failed in 10 cases (5 LCA and 5 RCA), and the success rate of bilateral coronary angiography in the STGC group was similar to that in the control group (97.1% vs. 96.4%, *p* = 0.34).

During the PCI procedure, nine patients required guiding catheter exchange (6 LCA and 3 RCA) in the STGC group, while 12 patients required guiding catheter exchange (5 LCA, 12 RCA) in the control group. The success rate of the initial guiding catheter for culprit vessel PCI was similar between the STGC and control groups (95.4% vs. 93.9%, *p* = 0.45). The total STGC success rate (both coronary angiography and culprit vessel PCI) in the STGC group was 92.5% (Supplementary Figure 1).

Eight patients in the STGC group and 12 patients in the control group crossed to the femoral route (*p* = 0.362). The causes of crossover in the STGC group were radial puncture failure (*n* = 1), radial/brachial tortuosity (*n* = 2), and tortuous innominate artery/subclavian artery (*n* = 5), while the causes in the control group were radial artery puncture failure (*n* = 4), radial/brachial tortuosity (*n* = 3), and right subclavian/aortic arch tortuosity (*n* = 5) (Supplementary Figure 1).

An ECG can identify LAD as the culprit vessel in anterior STEMI (100%). However, 13 patients (4.6%) in the STGC group with inferior STEMI failed to identify the culprit vessel by ECG (11 LCX and 1 LAD). Conversely, one patient was found to have an LCX as the culprit vessel that was missed on the ECG.

There were 22 (7.9%) patients in the STGC group and 20 (7.1%) patients in the control group who underwent non-culprit vessel PCI at the time of primary PCI. No emergency coronary artery bypass graft was performed in either of the groups (Table 2).

**TABLE 2:** Procedural characteristics.

	STGC group	Control group	*P*-value
	(*n* = 280)	(*n* = 280)	
Radial puncture failure, *n* (%)	1 (0.4)	4 (1.4)	0.37
Crossover to femoral, *n* (%)	8 (2.9)	12 (4.3)	0.36
Tirofiban, *n* (%)	15 (5.4)	14 (5.0)	0.85
**Number of diseased vessels, *n* (%)**			0.74
1	72 (25.7)	79 (28.2)	
2	82 (29.3)	83 (29.6)	
3	126 (45.0)	118 (42.1)	
**Culprit artery, *n* (%)**			0.70
LM	1 (0.4)	0	
LAD	129 (46.1)	127 (45.4)	
LCX	29 (10.4)	30 (10.7)	
RCA	121 (43.2)	123 (43.9)	
**TIMI flow before PCI, *n* (%)**			0.33
0/1	222 (79.3)	235 (83.9)	
2	17 (6.1)	15 (5.4)	
3	41 (14.6)	30 (10.7)	
**Initial guiding catheter, *n* (%)**			
LCA as culprit vessel	159	152	
JL3.5	137 (86.2)	86 (56.6)	<0.001
MAC3.5	22 (13.8)	12 (7.9)	0.09
Other	0 (0)	54 (35.5)	
RCA as culprit vessel	121	128	
JL3.5	94 (77.7)	28 (21.9)	<0.001
MAC3.5	27 (22.3)	17 (13.3)	0.062
Other	0 (0)	83 (64.8)	<0.001
**Type of primary PCI, *n* (%)**			0.07
PTCA	23 (8.2)	36 (12.9)	
Stent	257 (91.8)	244 (87.1)	
Aspiration catheter use, *n* (%)	236 (84.3)	234 (83.6)	0.82
Predilatation, *n* (%)	227 (81.1)	221 (78.9)	0.53
Postdilatation, *n* (%)	208 (74.3)	204 (72.9)	0.70
Stent length (mm)	35.6 ± 21.5	34.6 ± 24.2	0.60
Stent size (mm)	3.1 ± 0.9	3.0 ± 1.1	0.10
No. of stents implanted/patient	1.4 ± 0.8	1.4 ± 0.9	0.34
IABP, *n* (%)	17 (6.1)	16 (5.7)	0.86
Non-culprit vessel PCI (%)	22 (7.9)	20 (7.1)	0.75
**TIMI flow after PCI, *n* (%)**			0.42
0/1	4 (1.4)	6 (2.1)	
2	17 (6.1)	24 (8.6)	
3	259 (92.5)	250 (89.3)	
Procedural success, *n* (%)	266 (95.0)	261 (93.2)	0.37
Contrast volume, ml	138.6 ± 49.3	135.0 ± 50.9	0.39
**Initial catheter success for CAG**			
RCA success	275 (98.2)	275 (98.2)	1.00
LCA success	277 (98.9)	275 (98.2)	0.72
Catheter change during CAG, *n* (%)	8 (2.9)	10 (3.6)	0.63
Culprit vessel PCI GC change, *n* (%)	9 (3.2)	12 (4.3)	0.51
Number of guiding catheters	1.1 ± 0.4	1.2 ± 0.5	0.12
Total number of catheters*	1.2 ± 0.5	2.2 ± 0.6	<0.001
LCA culprit	1.2 ± 0.5	2.2 ± 0.5	<0.001
RCA culprit	1.2 ± 0.6	2.3 ± 0.7	<0.001

Values are mean ± SD, *n* (%). *Catheter include diagnostic catheter, guiding catheter, and guidezilla. CAG, coronary angiography; GC, guiding catheter; IABP, intra-aortic balloon pumping; LAD, left anterior descending artery; LCA, left coronary artery; LCX, left circumflex; LM, left main; PCI, percutaneous coronary intervention; PTCA, percutaneous transluminal coronary intervention; RCA, right coronary artery; TIMI, thrombolysis in myocardial infarction.

Eleven patients had radial artery angiography and were diagnosed with RAP when resistance was encountered during guiding catheter advancement (Supplementary Figure 2). RAP induced by advancing STGC was observed in two patients in the STGC group and nine patients during catheter exchange in the control group. The RAP rate was significantly higher in the control group than in the STGC group (3.2% vs. 0.7%, *p* = 0.033). The balloon-assisted tracking technique allowed the guiding catheter to pass through the site of perforation and PCI in 10 patients (two in the STGC group and eight in the control group), and radial artery angiography revealed that all the RAPs were sealed. Another patient in the control group switched to femoral access after guiding catheter-induced RAP, which was treated with external compression.

### Treatment time

**FIGURE 2:**
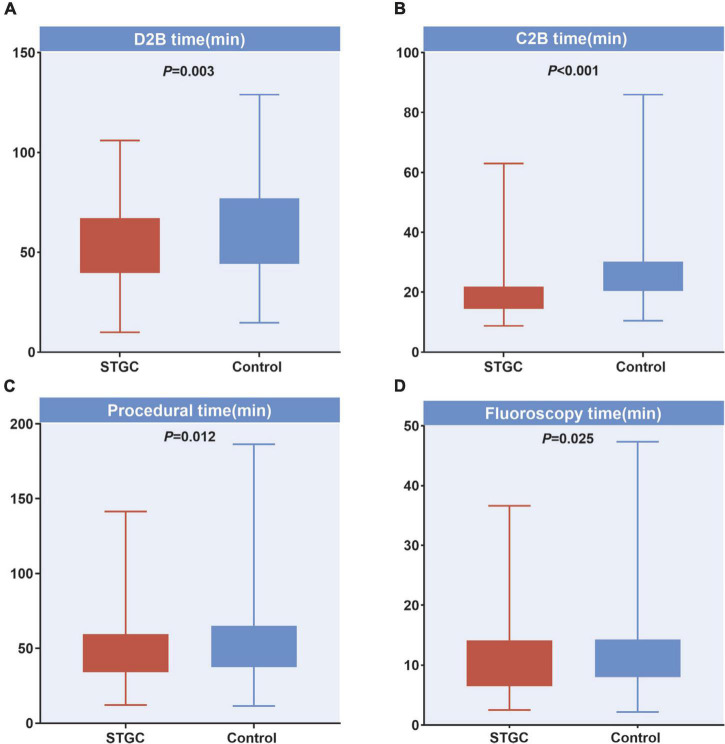
Difference of treatment time between two groups. **(A)** D2B time, **(B)** C2B time, **(C)** procedural time, and **(D)** fluoroscopy time. C2B, catheterization laboratory door-to-balloon; D2B, door to balloon; STGC, single transradial guiding catheter.

**FIGURE 3:**
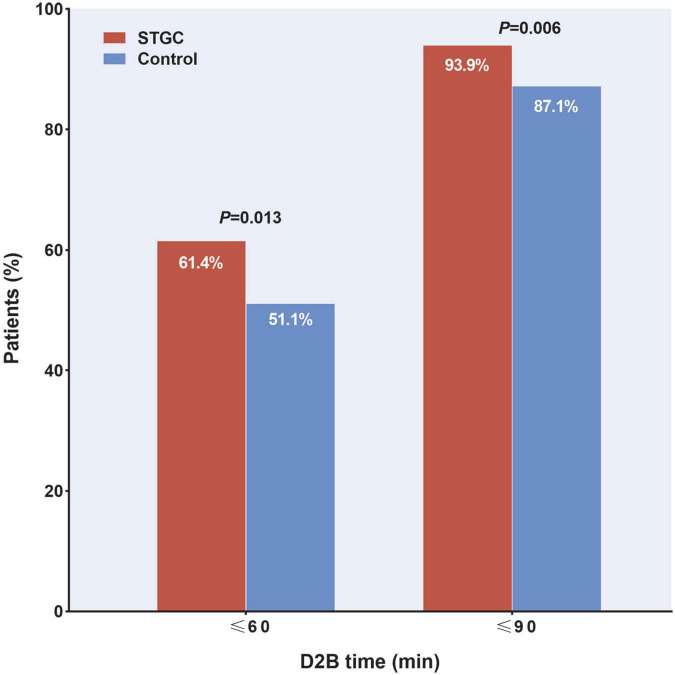
Proportion of patients achieving door-to-balloon (D2B) target. Proportion of patients with D2B time within 90 and 60 min were significantly higher in the STGC group than in control group (*p* = 0.006 and *p* = 0.013, respectively). D2B, door-to-balloon; STGC, single transradial guiding catheter.

**FIGURE 4:**
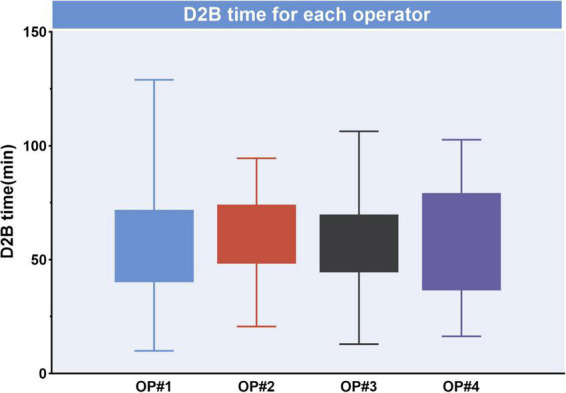
Median door-to-balloon (D2B) time for each operator. D2B was not significantly different between operators (*p* > 0.05), D2B, door-to-balloon.

**FIGURE 5:**
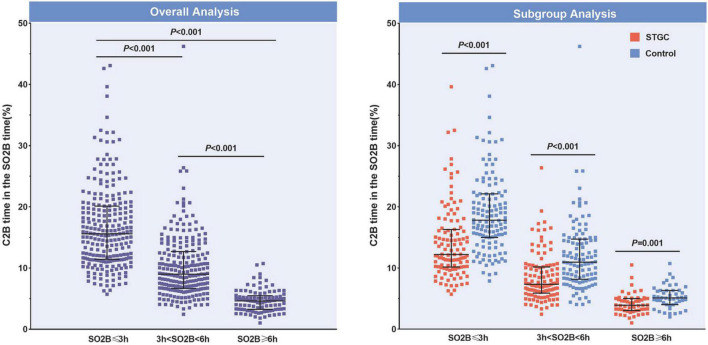
Proportions of C2B time in the SO2B time. The proportion of C2B time in the SO2B time was significantly different for patients with SO2B time ≤3, 3–6, and ≥6 h (15.6% vs. 9.0% vs. 4.6%, *p* < 0.001, respectively). In subgroup analysis, the proportion was also significant different between the STGC group and the control group (*p* < 0.001, respectively). C2B, catheterization laboratory door-to-balloon; SO2B, symptom to balloon; STGC, single transradial guiding catheter.

The median D2B (53.9 vs. 58.4 min, *p* = 0.003), C2B (17.3 vs. 24.5 min, *p* < 0.001), procedural (45.2 vs. 49.0 min, *p* = 0.012), and fluoroscopy times (9.7 vs. 11.3 min, *p* = 0.025) were significantly shorter in the STGC group compared with the control group (Figure 2 and Table 3). Per-protocol analyses come to essentially the same conclusions (Supplementary Table 1). The proportion of patients achieving D2B time within 90 and 60 min increased significantly in the STGC group (93.9% vs. 87.1%, *p* = 0.006; 61.4% vs. 51.1%, *p* = 0.013, respectively; Figure 3). There was no difference in the D2B time between the four operators (Figure 4). The proportion of C2B time in the symptom-to-balloon time was significantly higher for patients with symptom-to-balloon time ≤3, 3–6, and ≥6 h (15.6% vs. 9.0% vs. 4.6%, *p* < 0.001, respectively) and subgroup patients (Figure 5).

**TABLE 3:** Treatment times.

ITT analysis	STGC group	Control group	*P*-value
	(*n* = 280)	(*n* = 280)	
Procedural time, min	45.2 (34.1–59.5)	49.0 (37.5–65.0)	0.012
Fluoroscopy time, min	9.7 (6.5–14.1)	11.3 (8.0–14.3)	0.025
FMC2B time, min	79.3 (60.8–112.3)	78.0 (62.6–113.0)	0.65
D2C time, min	36.0 (21.1–48.8)	33.2 (20.0–50.0)	0.59
C2B time, min	17.3 (14.4–21.8)	24.5 (20.4–30.2)	<0.001
P2B time, min	10.5 (8.0–14.5)	16.8 (13.3–22.0)	<0.001
D2B time, min	53.9 (39.7–67.1)	58.4 (44.1–77.1)	0.003
D2B ≤ 90 min	263 (93.9)	244 (87.1)	0.006
D2B ≤ 60 min	172 (61.4)	143 (51.1)	0.013

Values are median (25th, 75th percentiles) or *n* (%). C2B, catheterization laboratory door-to-balloon; D2B, door to balloon; D2C, hospital door to catheterization laboratory; FMC2B, first medical contact to balloon; P2B, puncture-to-balloon; ITT, intention to treat analysis.

### Clinical outcomes

No catheter-associated coronary complications were observed in either group. There was no significant difference in MACE at 30 days (2.5% vs. 4.6%, *p* = 0.17) between the two groups (Table 4). After discharge, 17 patients in the STGC group and 14 in the control group underwent staged PCI.

**TABLE 4:** Clinical outcomes.

	STGC group	Control group	*P*-value
	(*n* = 280)	(*n* = 280)	
**In hospital clinical outcomes**			
In hospital MACE	7 (2.5)	8 (2.9)	0.79
Cardiac death	6 (2.1)	8 (2.9)	0.59
Reinfarction	1 (0.4)	1 (0.4)	1
TVR	1 (0.4)	1 (0.4)	1.00
Stroke	0	1 (0.4)	0.50
**30 days clinical outcomes**			
30 days MACE	7l(2.5)	13 (4.6)	0.17
Cardiac death	6 (2.1)	10 (3.6)	0.31
Reinfarction	1 (0.4)	2 (0.7)	1.00
TVR	1 (0.4)	3 (1.1)	0.62
Stroke	0	1 (0.4)	0.50
BARC bleeding ≥ 3 type	2 (0.7)	1 (0.4)	1.00
Staged PCI	17 (6.1)	14 (5.0)	0.58

Values are *n* (%). BARC, bleeding academic research consortium; MACE, major adverse cardiac events; PCI, percutaneous coronary intervention; TVR, target vessel revascularization.

## Discussion

Our study showed that D2B time and C2B time in the STGC group were significantly reduced by 4.4 and 7.2 min, respectively. This reduction translates to a higher proportion of cases with a D2B time of less than 90 min (93.9%). Procedural and fluoroscopy times were also significantly shorter in the STGC group. RAP occurred less frequently in the STGC group vs control group (3.2% vs. 0.7%, *p* = 0.03). There were no significant differences in MACE at 30 days (2.5% vs. 4.6%, *p* = 0.17) between the two groups.

### Decreasing door-to-balloon time

The survival benefit of primary PCI depends on the timely opening of the culprit vessel within the recommended 90 min or less D2B time and radial access first according to the current guidelines (1, 14). A shorter D2B time for individual patients is associated with lower mortality in hospitals (2, 15, 16), at 30 days (2, 16), at 6 months (17), or 1 year (2, 3). There has been extensive research on strategies to improve D2B time, including shortening the door to ECG and ECG to activation time (18), prehospital activation and direct to the cath lab (19, 20), and data feedback with each member in the system of care (20). However, little attention has been paid to the effect of the sequence of the catheter on D2B time in transradial access.

### Choice of the catheter on door-to-balloon time

One way to shorten D2B time is to perform transradial coronary angiography using a STGC. Several randomized trials have investigated the performance of either standard Judkins-shaped or dedicated catheters (Tiger II, Kimny, and DxTerity) for transradial coronary angiography (21–23). The potential objective of introducing the STGC method of transradial intervention is to avoid catheter exchange and achieve shorter procedural times. A recent meta-analysis showed comparable results for either dual- or single catheter strategies for transradial coronary angiography (23). It is unclear whether an STGC strategy for diagnostic coronary angiography is a time-saving measure in the setting of acute coronary syndrome.

Another way to shorten the D2B time is to use a STGC for both non-culprit and culprit vessel angiography and intervention. Several guiding catheters (Kimny, Ikari Left, RM, and MAC) have been studied during primary PCI (9–11, 24–27). In this strategy, an STGC is used to perform contralateral coronary angiography, followed by complete culprit vessel angiography and intervention. In a randomized study by Guo et al. (9), compared with conventional methods (universal diagnostic Tiger catheter followed by guiding catheter), using a single MAC3.5 guiding catheter significantly reduced the median procedure time by 3.4 min, and the D2B time was similar between the two groups. In another multicenter retrospective study, D2B time was significantly reduced by 6.0 min in the STGC group compared with the conventional group (10).

### Culprit vessel percutaneous coronary intervention first on door-to-balloon time

Data obtained in previous retrospective studies (12, 28) using an ECG-guided culprit-vessel first strategy before contralateral or complete diagnostic angiography indicated that a lower D2B time was achieved by skipping several steps compared with the conventional procedure. According to Couture et al. (12), the median vascular access-to-balloon time was 4–6 min shorter with a culprit-vessel PCI strategy. Plourde et al. (28) reported that the median D2B time in the immediate PCI group was 8 min less than that in the control group (32 vs. 40 min, *p* < 0.0001). In the present randomized study, ECG-guided culprit vessel-first PCI enabled the improvement of D2B and C2B by 4.4 and 7.2 min, respectively, which was similar to the above reports (12, 28). In addition, in our study, the same guiding catheter was also applied to perform the contralateral angiography after culprit vessel-PCI, which resulted in a shorter fluoroscopy time by 1.6 min and procedure time by 4.0 min compared to a standard approach. These results are also consistent with our previous study (9). Furthermore, no guiding catheter-induced coronary complications were observed in either group.

### Potential benefits for radial access

Radial artery spasm is the most frequent complication of transradial cardiac catheterization. Previous studies (23, 29) have shown that the number of catheters used is associated with radial artery spasm. RAP is extremely rare, occurring in <1% of cases, and may occur as a consequence of guidewire advancement and avulsion due to catheter advancement (29). In the present study, we did not calculate the variable of radial artery spasm, but we found that the rate of RAP was significantly lower in the STGC group than in the control group (0.7% vs. 3.2%, *p* = 0.03). All the RAPs occur at the time of guiding catheter advancement, in the nine cases of the control group, spasmolytic agents were not administered intra-arterially before catheter exchanges to avoid spasm, which may the reason of RAPs (22). Therefore, spasmolytic agents should be used during catheter exchange.

### Important questions that need to be focused on

In contrast to traditional approach, our new strategy proposed here allows issues such as failure to identify the culprit artery by ECG in inferior STEMI, emergency coronary artery bypass graft, and time-saving clinical implications to be considered.

Identification of the culprit artery in inferior STEMI plays an important role in the culprit vessel first strategy; misdiagnosis of culprit vessels may delay C2B time. In our study, the diagnostic accuracy was 95.4%, which is consistent with results obtained in previous studies (30, 31). Owing to the use of a STGC strategy, the STGC can be easily manipulated to engage the contralateral artery without a time delay.

It is important to consider that diagnostic angiography before PCI might provide valuable anatomical information that could change the reperfusion strategy, such as an emergency coronary artery bypass graft. However, coronary artery bypass graft was performed as reperfusion therapy in only 0.8% of patients (32), and patients requiring urgent or emergency coronary artery bypass graft within 24 h of STEMI have mortality rates of 8.2∼15.8% (33, 34). In fact, for patients with STEMI and multivessel disease, immediate or staged complete revascularization with PCI significantly improves hard clinical outcomes (35). In our study, immediate complete revascularization was performed in 7.5% of the patients (contralateral 4.3%, ipsilateral 3.2%) in the STGC group and 6.8% in the control group (contralateral 4.6%, ipsilateral 2.2%), and there was no coronary artery bypass graft in the acute period and 30 days after PCI.

Previous studies have demonstrated that every minute of delay in primary PCI affects 1 year mortality (36). Recently, Park et al. (3) showed that reduction in D2B time by 30 min was associated with a continuous reduction in 1 year mortality. However, population-level studies revealed that reducing D2B time in primary PCI did not improve mortality (17, 37). These results raise questions regarding the value of shortening D2B time. In our study, a reduction of 4.4 min D2B time failed to translate into clinical outcomes, which is in agreement with previous studies (12, 38). The following possible reasons should be considered: (1) the primary endpoint is D2B time other than symptom to balloon time in our study, and clinical outcome differences cannot be identified due to the small sample size. (2), the C2B time represents only a small fraction of the symptom to balloon time. In our study, the C2B time encompassed 15.6% of the symptom to balloon time in patients with symptom to balloon time ≤3 h. Improvement in C2B time might have a significant effect on the reduction of symptom to balloon time, especially in patients presenting within 3 h of symptom onset. (3) “Time is muscle” remains indisputable. Time may also be critical in certain clinical situations, for example, in a patient with cardiogenic shock (39). (4) The contribution of radial access to better outcomes may cause bias in the interpretation of the relationship between our time-saving strategy and the outcomes.

To our knowledge, this is the first randomized study to investigate the effectiveness of two-strategy combination of using a STGC and culprit vessel-PCI first. Our results provide compelling evidence for this new strategy with shorter D2B, C2B, fluoroscopy, and procedure times.

## Study limitations

Our study has several limitations. First, this was an open-label trial, which may have caused potential bias in the results. Second, this study was conducted at a high-volume tertiary center for primary PCI. Therefore, the results may not be generalizable to low-volume primary PCI centers. Third, the right radial artery is the default access. MAC3.5 or JL3.5 manipulation technique may not apply to the left radial access. Fourth, the primary endpoint was D2B time, but not clinical endpoint. Fifth, although prasugrel is not available in China, the use of ticagrelor remains low, only 52.9% of patients were under ticagrelor treatment in the present study, highlighting a therapeutic inertia with considerable gap between guidelines and clinical practice. Sixth, there was a higher proportion (>80%) of male in the current study, may not be representative of acute coronary syndrome in usual clinical practice. Seventh, aspiration thrombectomy were performed in a high rate of patients in current study. However, there was no significant difference between the two groups, which would not be expected to influence the primary endpoint. Eighth, low incidence of AF patients were detected. Finally, the follow-up duration in this study was relatively short. Additional longer follow-up should be performed to evaluate clinical outcomes.

## Conclusion

ECG-guided immediate intervention on the culprit vessel followed by contralateral angiography with a STGC in STEMI patients is safe and is associated with a 4.4 min decrease in D2B time, 7.2 min in C2B time, reduced procedural time, fluoroscopy time, and RAP complications. Further studies are required to evaluate the effects of this strategy on the clinical outcomes.

## Data availability statement

The datasets used and/or analyzed during this current study are available from the corresponding authors on reasonable request. Requests to access these datasets should be directed to JCG, guojcmd@126.com.

## Ethics statement

The studies involving human participants were reviewed and approved by the Review Board of Beijing Luhe Hospital. The patients/participants provided their written informed consent to participate in this study.

## Author contributions

JCG and QH: conception, design, final approval of the manuscript, and overall responsibility. ZJL, YJW, SHW, YTW, YXW, HTW, YPW, and LBZ: data collection. JCG, GZW, and ZXL: analysis, interpretation, and manuscript writing. QH: critical revision. JCG: obtain funding. All authors contributed to the article and approved the submitted version.

## Abbreviations

C2B: catheterization laboratory door-to-balloon
D2B: door-to-balloon
LAD: left anterior descending artery
LCX: left circumflex artery
MACE: major adverse cardiac events
PCI: percutaneous coronary intervention
RAP: radial artery perforation
RCA: right coronary artery
STGC: single transradial guiding catheter
STEMI: ST-elevation myocardial infarction.
