# Supplementary material for: Culprit vessel revascularization first with primary use of a dedicated transradial guiding catheter to reduce door to balloon time in primary percutaneous coronary intervention
Source: Front Cardiovasc Med. 2022 Oct 28;9:1022488. doi: 10.3389/fcvm.2022.1022488 (PMC9649753; doi:10.3389/fcvm.2022.1022488)
Supplement: Supplementary file 1 [file Data_Sheet_1.docx]

**Supplementary Figure 1 Flowchart of catheter success**


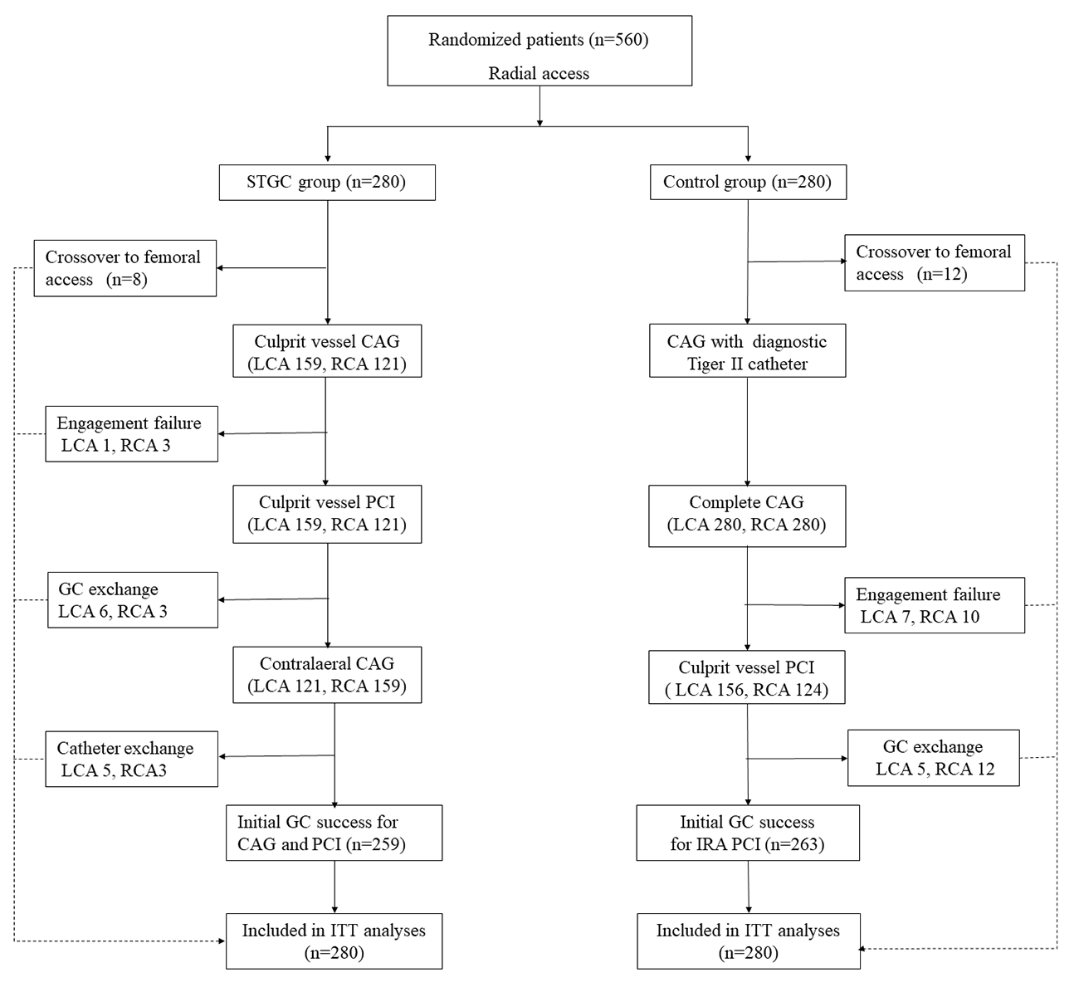


CAG=coronary artery angiography; CVA=culprit vessel angiography; GC=guiding catheter; ITT=intention to treat analysis; LCA=left coronary artery; RCA=right coronary artery; PCI=percutaneous coronary intervention; STGC=single transradial guiding catheter.

**Supplementary Figure 2 Radial perforation rate**


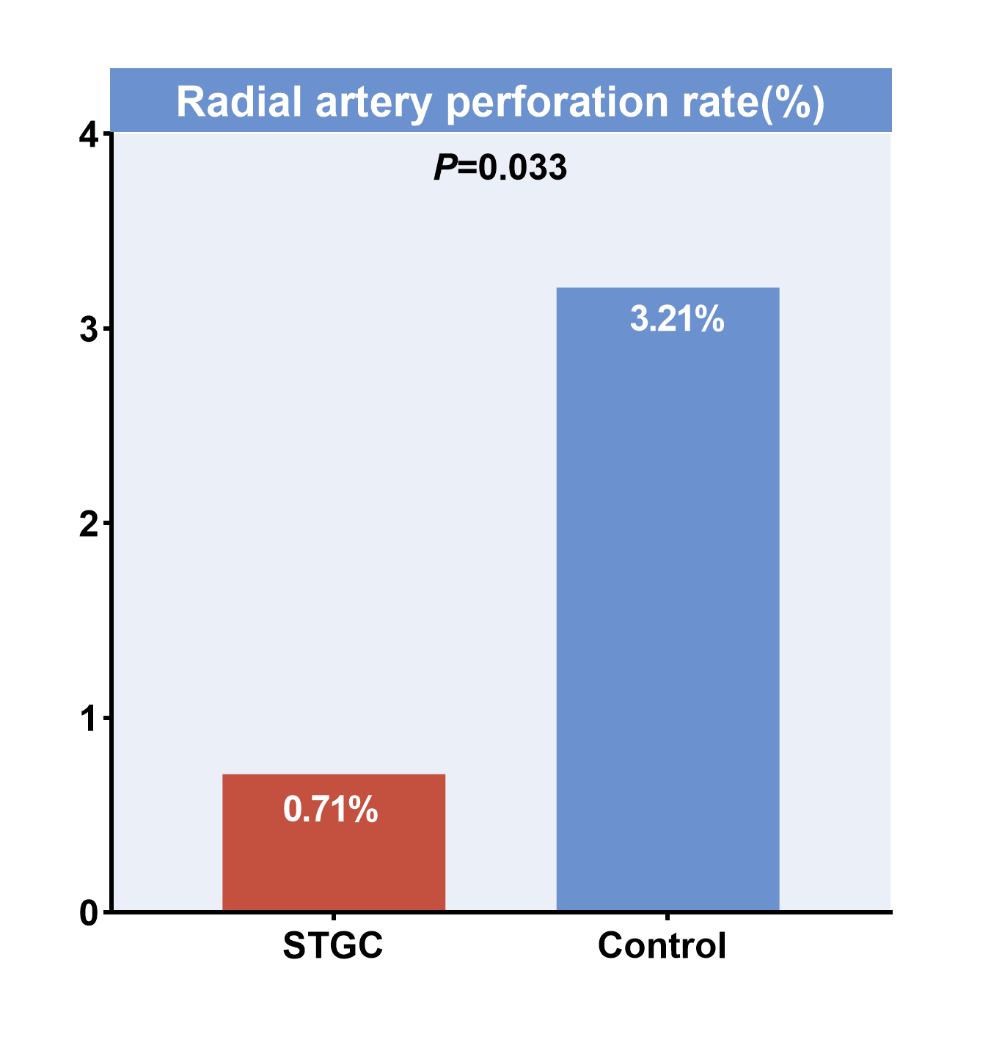


Bar graphs denoting radial perforation rate were significantly reduced in the STGC group compared with the control group.

**Supplementary Video 1 Manipulation of JL 3.5 guiding catheter for RCA**

JL 3.5 is advanced to the floor of right coronary sinus with the standard wire in the secondary curve of the catheter, which is rotated clockwise to cannulate the RCA ostium.

RCA=right coronary angiography

**Supplementary Video 2 Manipulation of MAC 3.5 guiding catheter**

Manipulation of the MAC3.5 guiding catheter for engagement of right coronary artery is like Judkins Right traditionally used (Video 2A). For left coronary artery, advancing MAC 3.5 against the contralateral aortic wall to form a U-shape, rotate the catheter clockwise toward the left sinus to cannulate the left ostium (Video 2B).

| **Supplementary Table 1** **Treatment times** | | | |  |
| --- | --- | --- | --- | --- |
|  | **STGC group** | **Control group** |  |  |
| **PP analysis** | **(n=272)** | **(n=268)** | **p Value** |  |
| Procedural time, min | 44.0(34.0-59.1) | 48.5(36.9-64.0) | 0.016 |  |
| Fluoroscopy time, min | 9.4(6.4-13.4) | 10.6(7.5-13.5) | 0.04 |  |
| FMC2B time,min | 78.6(60.4-111.9) | 79.4(62.4-111.5) | 0.60 |  |
| D2C time, min | 36.0(23.0-49.0) | 33.0(18.6-50.0) | 0.30 |  |
| C2B time, min | 17.0 (14.4-21.5) | 24.3(20.3-29.0) | <0.001 |  |
| P2B time,min | 10.2(8.0-13.7) | 16.0(13.0-20.6) | <0.001 |  |
| D2B time, min | 53.6(39.1-66.6) | 58.0(44.1-75.8) | 0.005 |  |
| D2B≤90 min | 256(94.1) | 236(88.1) | 0.013 |  |
| D2B≤60 min | 169(62.1) | 141(52.6) | 0.025 |  |
| Values are median (25th,75th percentiles) or n (%).  C2B=catheterization laboratory to balloon; D2B=door to balloon; D2C=hospital door to catheterization laboratory; FMC2B=first Medical Contact to Balloon; P2B=puncture-to-balloon; PP=per protocol. | | | |  |
|  |  |  |  |  |
|  |  |  |  |  |
